# Can anti-bothropstoxin-I antibodies discriminate between *Bothrops jararaca* and *Bothrops jararacussu* venoms?

**DOI:** 10.1186/s40409-017-0105-z

**Published:** 2017-03-11

**Authors:** Ricardo Teixeira Araujo, Carlos Corrêa-Netto, Leonora Brazil-Más, Caio Raony Farina Silveira, Irene Fernandes, Russolina Benedeta Zingali

**Affiliations:** 1 Laboratório de Hemostase e Venenos, Instituto de Bioquímica Médica Leopoldo de Meis, Universidade Federal do Rio de Janeiro (UFRJ), Rio de Janeiro, RJ Brasil; 2 Instituto Vital Brazil, Niterói, RJ Brasil; 3 Instituto Butantan, São Paulo, SP Brasil

**Keywords:** *Bothrops jararacussu*, *Bothrops jararaca*, Bothropstoxin-I, Monoclonal antibodies, Species specificity

## Abstract

**Background:**

Snakes of the genus *Bothrops*, popularly known as pit vipers, are responsible for most cases of snakebite in Brazil. Within this genus, *Bothrops jararacussu* and *B. jararaca* deserve special attention due to the severity of their bites and for inhabiting densely populated areas. Regarding the treatment of snakebites by *Bothrops jararacussu*, questions have been raised about the effectiveness of the specific bothropic antivenom in neutralizing myotoxic effects; however, there are no accurate data for humans. Thus, the development of a differential diagnostic kit for this species would be of great interest because it provides, for healthcare professionals, a tool that would allow us to determine whether the accident was caused by *B. jararacussu* or other species of the genus. It would also make it possible to evaluate the specificity of the treatment and to provide data for epidemiological studies.

**Methods:**

First, we produced a species-specific polyclonal antibody – a potential biomarker of *Bothrops jararacussu* venom – against bothropstoxin-I (BthTx-I), which is also found in smaller quantities in the venoms of *B. jararaca* from southern Brazil.

**Results:**

Polyclonal antibodies against bothropstoxin-I could be separated into several species-specific immunoglobulins. Then, aiming to develop a system of safe and standardized immunoassay, we produced monoclonal antibodies. Seven hybridomas were obtained. Five of them were specific to the venom of *B. jararacussu* and two recognized the venom of *B. jararaca* from the southeastern population. The use of monoclonal antibodies also made it possible to differentiate *B. jararacussu* from *B. jararaca* venom obtained from the southern population. Analyzing the reactivity of monoclonal antibodies against other bothropic venoms, we found mAb Bt-3 to be more specific than others for *B. jararacussu* venom.

**Conclusions:**

These results show the potential of BthTx-I for producing monoclonal antibodies that differentiate between *B. jararacussu* and other *Bothrops* species venoms.

## Background

Among the snake species of the genus *Bothrops*, *Bothrops jararacussu* is remarkable for the low immunogenicity of its venom, which is highly myotoxic, leading to necrosis of striated muscle fibers and slowing tissue regeneration [1, 2]. In all Brazilian states where *B. jararacussu* is present, *B. jararaca* is also found [3]. This makes it difficult to differentiate between these two snakes when an accident occurs, due to extensive homology among envenomation symptoms [4, 5]. In Rio de Janeiro, both species have clinical importance. In the envenomation context, the question of the efficiency of specific bothropic antivenom against *B. jararacussu* bites is often discussed, since the myotoxic effects observed in mice are not completely neutralized. For this reason, some researchers have suggested the use of a combinated bothropic-crotalic antivenom as a more appropriate treatment [1, 6–10].

The proteomic characterization of *B. jararacussu* venom and the immunoreactivity of anti-*B. jararaca* and anti-*B. jararacussu* sera have contributed to our understanding of some of the immunochemical characteristics of *B. jararacussu* venom and led to a suggestion for the use of bothropstoxin-I (BthTx-I) as a biomarker molecule [11]. This molecule is a phospholipase A_2_ Lys-49 (Lys-49 PLA_2_) from *B. jararacussu* venom with three α-helices and two antiparallel β-sheets [12, 13]. This protein is the most abundant myotoxin that can be isolated from *B. jararacussu* venom and although it exhibits severe neurotoxicity and myotoxicity, it shows little or no phospholipase activity [13, 14]. This toxin is able to promote injury in lipid bilayer of cell membranes through a calcium-independent mechanism, inducing myonecrosis [13, 15].

Lys49-PLA_2_ molecules have been described in various *Bothrops* venoms, such as BnSP-7 from *B. neuwiedi*, myotoxin I from *B. moojeni*, and BaTX from *B. alternatus*, among others [16–18]. Recently, Gonçalves-Machado et al. [19] described the presence of BthTx-I in venoms of *B. jararaca* from the southern region of Brazil. Methods for differentiating the envenomation caused by *B. jararacussu* from the one caused by *B. jararaca* are very important in order to enable the epidemiological study of accidents with these two snakes, as well as to allow studies of treatment efficiency for *B. jararacussu* bites in humans. In this context, the objective of the present study was to produce monoclonal antibodies from BthTx-I to be used as tools for the development of a differential diagnostic kit for bites provoked by *B. jararacussu.*

## Methods

### Animals and venoms

BALB/c mice (18–20 g) were provided by the Butantan Institute. All procedures were approved by the Ethics Committee for Animal Research of Butantan Institute (process no. 697/10). The venoms of *Bothrops alternatus, B. atrox, B. diporus, B. erytromelas, B. fonsecai, B. insularis, B. jararaca, B. jararacussu, B.leucurus, B. moojeni, B. neuwiedi* and *B. pubescens* were provided by the Laboratory of Herpetology of the Vital Brazil Institute.

### Isolation of bothropstoxin-I

Bothropstoxin-I was isolated following the description of Correa-Netto et al. [11]. Briefly, *B. jararacussu* venom (500 mg) dissolved in 5 mL buffer (20 mM Tris-HCl, 150 mM NaCl, pH 8.8) was applied onto a Sephacryl S-200 HR column (2.6 × 100 cm) with a flow rate of 17 mL/h generating four distinct peaks. The third peak, which contained proteins with a maximal apparent molecular mass of 30 kDa, was dialyzed against PBS buffer (50 mM sodium phosphate/150 mM sodium chloride, pH 7.4) and applied to an ion-exchange column (Mono S HR 5/5, Pharmacia) at a flow rate of 1.0 mL/min. Elution using a linear gradient of 0–1 M NaCl in the same phosphate buffer yielded two peaks; the second was identified as BthTx-I by mass spectrometry. The elution profile was monitored by absorbance at 280 nm.

### Polyclonal anti-BthTx-I serum produced in rabbits

Two rabbits were injected subcutaneously with 500 μg of BthTx-I emulsified in complete Marcol/Montanide adjuvant. After the first injection, boosters were made 2, 3, 4 and 5 weeks later with incomplete Marcol/Montanide adjuvant. Blood samples were drawn after the 5th week and the immune serum was collected.

### Purification of polyclonal species-specific antibodies

Two columns of Sepharose 4B activated by cyanogen bromide were prepared, one with *Bothrops jararacussu* (jararacussu-Sepharose) and the other with *Bothrops jararaca* from the southeast region of the country (jararaca-Sepharose). The column preparation followed instructions from Amersham Biosciences. Both columns were equilibrated with PBS buffer (50 mM sodium phosphate/150 mM sodium chloride, pH 7.4). Anti-BthTx-I serum was applied to the jararacussu-Sepharose column at a flow rate of 1 mL/min, thereafter discarding the unbound material. Immunoglobulins with affinity for the *B. jararacussu* venom were collected and applied to the jararaca-Sepharose column at a flow rate of 1 mL/min. The immunoglobulins that did not bind to the column were collected and those with affinity for the venom of *B. jararaca* were discarded. The pool of IgGs that did not bind to the column was designated “purified species-specific anti-BthTx-I”. For the elution of immunoglobulins from both columns, 0.2 M glycine/HCl buffer, 0.15 M NaCl, pH 2.8 was used. The elution was monitored by absorbance at 280 nm.

### Immunization of mice

Mice (female Balb/c) were immunized according to Fernandes et al. [20]. Four days after the last booster, animals were bled from the ophthalmic plexus and one animal immunized with BthTx-I was killed and popliteal lymph nodes were removed.

### Production and purification of monoclonal antibodies (mAbs)

Monoclonal antibodies were produced as described by Köhler and Milstein [21], with modifications. Popliteal lymph node cells from BALB/c mice immunized with BthTx-I were fused with SP2-O cells (2:1) using polyethylene glycol 4000 (Merck). Hybrids were selected in RPMI 1640 medium containing 3% HAT (hypoxanthine 10 mM, aminopterin 40 mM and thymidine 1.6 mM; Gibco-BRL) and 10% FCS (Gibco-BRL) at 37 °C and 5% CO_2_. Supernatant fluids were screened for species-specific antibodies by ELISA, as described in the next section. Antibody-secreting cells were expanded and cloned twice at limiting dilution. The mAbs contained in culture supernatants were purified by affinity chromatography on protein-A Sepharose (Pharmacia) equilibrated in borate saline buffer, pH 8.5. The proteins were eluted in 0.2 M glycine/HCl buffer, 0.15 M NaCl, pH 2.8, and dialyzed in borate saline buffer. An isotyping kit (Sigma) was used to determine the heavy-chain isotype.

### ELISA

ELISA was carried out according to Theakston et al. [22]. Briefly, wells were coated with venom (1 μg/well) or BthTx-I and, after blocking with 3% bovine serum albumin, various dilutions of mAbs were added to a final volume of 100 μL. Antigen–antibody reaction was detected by addition of anti-mouse IgG-peroxidase conjugate and ortho-phenylenediamine (1 mg/mL, Sigma) and H_2_O_2_ as enzyme substrates.

### Dot blot

Dot blot was carried out as described by Towbin et al. [23], with modifications. Briefly, samples (1 μg/μL) were applied directly to nitrocellulose membranes. After blocking with 3% bovine serum albumin, dilutions of mAbs were added. Antigen–antibody reaction was detected by addition of anti-mouse IgG-peroxidase conjugate and the reaction with a chromogenic substrate, 4-chloro-1-naphthol 0.05% in 15% methanol in the presence of 0.03% H_2_O_2_.

## Results

### Polyclonal species-specific antibodies

**Fig. 1:**
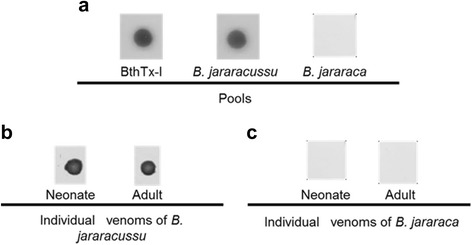
Species-specific recognition of anti-BthTx-I against *B. jararacussu* and *B. jararaca* venom. The purified antibodies were tested against (**a**) pools, individual venoms of (**b**) *B. jararacussu* and (**c**) southeastern *B. jararaca* of different ages (neonate and adults) through dot blot. One microgram of each venom was applied to a nitrocellulose membrane and subjected to recognition by the polyclonal species-specific antibodies at a dilution of 1:1000

We first tested whether the anti-BthTx-I polyclonal antibodies could give specific antibodies to recognize venoms from *B. jararacussu* and *B. jararaca*. For this purpose, we produced polyclonal antibodies in rabbits and tested against purified BthTx-I, *B. jararacussu* and *B. jararaca* (southeastern) venoms. As expected, the antibodies recognized both venoms when analyzed by ELISA and immunobloting (data not shown). With the goal of eliminating cross-reactive IgGs from polyclonal serum, these antibodies were subjected to affinity chromatography as described in Methods section, which yielded specific IgGs called here species-specific anti-BthTx-I. This set of antibodies clearly recognized *B. jararacussu* venom regardless of whether they were analyzed against individual or pooled venoms or against purified BthTx-I (Fig. 1) and did not recognize *B. jararaca* venom. These data clearly showed the utility of BthTx-I in generating such specific antibodies.

### Production and characterization of monoclonal antibodies (mAbs)

**Fig. 2:**
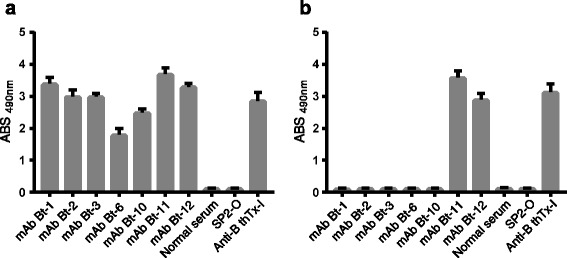
Analysis of mAb recognition of *Bothrops* venoms. ELISA plates were sensitized with crude venom of (**a**) *B. jararacussu* or (**b**) southeastern *B. jararaca*, and then tested with mAbs. The test was performed in triplicate and serum polyclonal anti-BthTx-I produced in mice was used as positive control. Normal serum and supernatant of SP2-O cells provided negative controls

Since polyclonal antibodies produced against BthTx-I were able to differentiate between the venom of two species, we decided to produce monoclonal antibodies. Fusion of myeloma SP2-O cells with popliteal lymphocytes of mice immunized with BthTx-I resulted in 354 hybridomas of which 21 secreted antibodies against BthTx-I. For cloning, we selected 12 hybridomas, tested by ELISA, that produced the highest optical density (>1.0) of antibodies; these were recloned to ensure monoclonality. Seven stable, immortalized clones secreting anti-BthTx-I antibodies were obtained. These mAbs were designated Bt-1, Bt-2, Bt-3, Bt-6, Bt-10, Bt-11 and Bt-12. All were mAbs belonging to the IgG1 isotype except mAb Bt-6, which belongs to the IgG2b isotype. The mAbs were analyzed by ELISA regarding their ability to recognize venoms from *B. jararacussu* and southeastern *B. jararaca*. The mAbs Bt-1, Bt-2, Bt-3, Bt-6 and Bt-10 showed specific reactivity for *B. jararacussu* venom while Bt-11 and Bt-12 could recognize both venoms (Fig. 2).

**Fig. 3:**
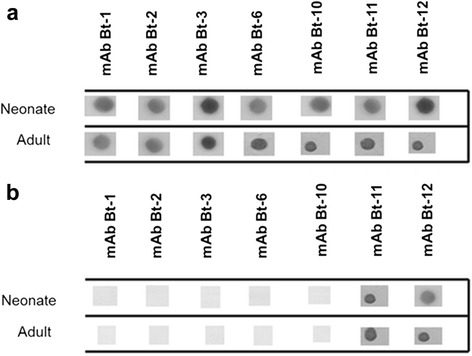
Analysis of mAbs against neonates and adult individual venoms. In (**a**) individual venom of *B. jararacussu* and (**b**) individual venom of southeastern *B. jararaca*. One microgram of each venom was applied to a nitrocellulose membrane and incubated with mAbs, followed by anti-mouse IgG-peroxidase. The antigen-antibody reaction was developed. Given the reproducibility of the mAbs against ten individual venoms (neonates and adults) of *B. jararacussu* and *B. jararaca*, in this study we show representative results using the venom of one neonate and one adult of each snake

Since ontogenetic and individual variations are described in *Bothrops* venoms we analyzed the specificity with which mAbs could recognize individual venoms of ten neonates and adults of *B. jararacussu* and southeastern *B. jararaca*. The mAbs Bt-1, Bt-2, Bt-3, Bt-6 and Bt-10 were specific for neonates and adults of *B. jararacussu* but did not recognize the venoms of southeastern *B. jararaca* (Fig. 3a). On the other hand, mAbs Bt-11 and Bt-12 recognized both venoms (*B. jararacussu and B. jararaca*) independently of the age (Fig. 3b).

### Analyzing the reactivity of mAbs against the venom *of B. jararaca* from the south of Brazil

**Fig. 4:**
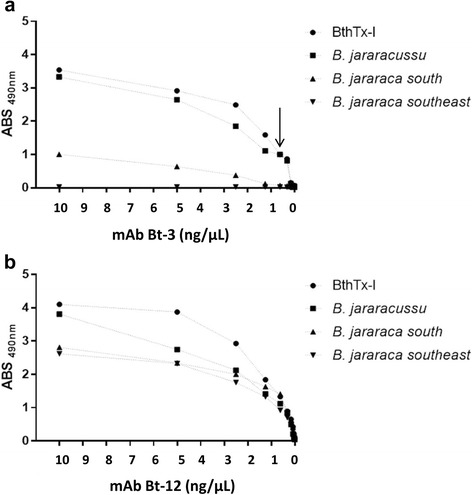
Titration of monoclonal antibodies. A 96-well plate was sensitized overnight with one microgram of BthTx-I and venoms from *B. jararacussu,* southeastern *B. jararaca* and southern *B. jararaca*. The plate was then incubated with various concentrations of mAb and revealed with anti-mouse IgG labeled with peroxidase. The arrow in (**a**) shows the concentration of mAb Bt-3 (0.62 ng/μL) which recognizes BthTx-I and *B. jararacussu* venom. The venoms from *B. jararaca* from the south and southeast were not recognized. In (**b**), titration of mAb Bt-12. Given the reproducibility of the mAbs against BthTx-I, *B. jararacussu,* southeastern *B. jararaca* and southern *B. jararaca* venoms, only a representative result is showed for mAb Bt-3 (representing results from Bt-1, Bt-2, Bt-6 and Bt-10) and for mAb Bt-12 (representing results from Bt-11)

As soon as we discovered the presence of BthTx-I in the venom of *B. jararaca* from the south region of Brazil, we evaluated its reactivity to mAbs using ELISA [15]. First, a serial dilution of mAbs in PBS was added. With a high mAbs concentration, venom of southern *B. jararaca* could be detected. However, as the mAbs concentration decreased, the intensity of the signal was gradually reduced until complete abrogation at 0.62 ng/μL. This result was reproduced with mAb Bt-1, Bt-2, Bt-3, Bt-6 and Bt-10. Hence, Bt-11 and Bt-12 do not lose reactivity to venom of southern *B. jararaca*, as expected. Figure 4 shows an example of the results obtained using Bt-3 and Bt 11.

**Fig. 5:**
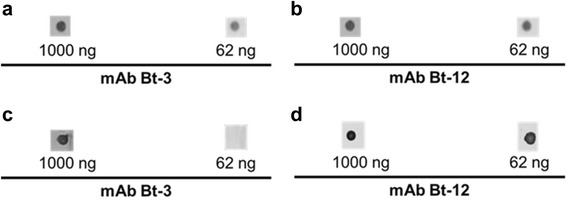
Analysis of mAbs against *B. jararacussu* and *B. jararaca* (south) venoms. One microgram of *B. jararacussu* or southern *B. jararaca* venom was applied directly to a nitrocellulose membrane. After blocking, 1000 or 62 nanograms of mAbs was applied. In (**a**) and (**b**) mAbs against *B. jararacussu* venom and (**c**) and (**d**) mAbs against southern *B. jararaca* venom. Given the reproducibility of the mAbs against the *B. jararacussu* and southern *B. jararaca* venoms, only a representative result for mAb Bt-3 (results from Bt-1, Bt-2, Bt-6 and Bt-10) and another for mAb Bt-12 (results from Bt-11) are showed

To confirm these data, a dot-blot analysis was carried out in order to compare effects of different mAbs and dilutions against venom from *B. jararacussu* and southern *B. jararaca*. It was again demonstrated that by using 62 nanograms of mAbs it is possible to detect venom from *B. jararacussu*, but not the one of *B. jararaca* from the south. At that concentration, only Bt-11 and Bt-12 were able to detect BthTx-I in both *B. jararacussu* and *B. jararaca* venom from the south (Fig. 5).

### Cross-reactivity with venoms from different species

We investigated whether these mAbs would recognize venoms of other *Bothrops* species from Brazil. The mAbs Bt-1, Bt-2, Bt-6, Bt-10, Bt-11 and Bt-12 showed cross-reactivity with the venom of *B. leucurus, B. moojeni, B. neuwiedi* and *B. pubescens.* However, Bt-3 was highly specific to the venom of *B. jararacussu,* not recognizing any other Brazilian *Bothrops* venom (Table 1). The mAbs Bt-10 and Bt-11 showed cross-reactivity with the venoms of *B. diporus* and *B. atrox* (Table 1). Dot blot confirmed the results obtained by ELISA (not shown). Monoclonal 3 (Bt-3) showed the highest specificity for *B. jararacussu* venom, highlighting the potential for this antibody as a tool for identifying accidents by *B. jararacussu* in some Brazilian states.

**Table 1:** Cross-reactivity with Brazilian *Bothrops* venoms by ELISA

	Bt-1	Bt-2	Bt-3	Bt-6	Bt-10	Bt-11	Bt-12	Anti-BthTx-I	NS
*Bothrops alternatus*	–	–	–	–	–	–	–	–	–
	(0.134)	(0.110)	(0.136)	(0.112)	(0.103)	(0.141)	(0.118)	(0.151)	(0.102)
*Bothrops atrox*	–	–	–	–	****	****	–	****	–
	(0.221)	(0.119)	(0.117)	(0.134)	(2.100)	(2.201)	(0.313)	(2.231)	(0.110)
*Bothrops diporus*	–	–	–	–	–	****	–	****	–
	(0.208)	(0.147)	(0.109)	(0.217)	(0.214)	(2,314)	(0.219)	(2.403)	(0.108)
*Bothrops erytromelas*	–	–	–	–	–	–	–	–	–
	(0.144)	(0.152)	(0.201)	(0.141)	(0.226)	(0.352)	(0.324)	(0.150)	(0.104)
*Bothrops fonsecai*	–	–	–	–	–	–	–	–	–
	(0.187)	(0.138)	(0.184)	(0.209)	(0.213)	(0.247)	(0.298)	(0.162)	(0.103)
*Bothrops insularis*	–	–	–	–	–	–	–	–	–
	(0.201)	(0.192)	(0.199)	(0.229)	(0.114)	(0.232)	(0.288)	(0.142)	(0.111)
*Bothrops jararaca* (southeast)	–	–	–	–	–	****	****	****	–
	(0.234)	(0.103)	(0.148)	(0.136)	(0.132)	(2.503)	(2.603)	(2.633)	(0.131)
*Bothrops jararacussu*	****	****	****	****	****	****	****	****	–
	(2.657)	(2.421)	(2.303)	(2.529)	(2.437)	(2.509)	(2.619)	(2.628)	(0.122)
*Bothrops leucurus*	****	**	–	****	****	****	–	****	–
	(1.407)	(0.634)	(0.132)	(1.914)	(2.098)	(2.001)	(0.215)	(2.383)	(0.134)
*Bothrops moojeni*	****	**	–	****	****	****	****	****	–
	(1.543)	(0.899)	(0.131)	(2.008)	(2.103)	(2.125)	(2.244)	(2.479)	(0.143)
*Bothrops neuwiedi*	****	**	–	****	****	****	–	****	–
	(1.722)	(0.987)	(0.124)	(2.103)	(2.103)	(2.136)	(0.200)	(2.501)	(0.127)
*Bothrops pubescens*	****	**	–	****	****	****	–	****	–
	(1.481)	(0.923)	(0.116)	(2.002)	(2.103)	(2.212)	(0.235)	(2.426)	(0.149)

Polyclonal anti-BthTx-I serum and normal serum (NS) of mice were utilized

**** Strong binding (optical density > 1.0); ** medium binding (optical density between 0.5 and 1.0); − weak or no binding (optical density between 0.1 and 0.5)

## Discussion

The use of immunodiagnostic tests that allow for the elucidation of the pattern of envenomation caused by venomous animals is recommended by the World Health Organization. This emphasizes the need to improve the quality of epidemiological and clinical data on accidents caused by venomous animals, in order to improve the therapeutic approach [24]. Nevertheless, common antigens present in venoms from different snake species have shown to be a major problem in developing immunodiagnostic tests [25–27].

In this study, we produced polyclonal antibodies in rabbits against BthTx-I with the aim of differentiating between venoms from *B. jararacussu* and *B. jararaca,* snakes of medical importance for the southeastern region of Brazil, especially the state of Rio de Janeiro. The produced antibodies showed extensive cross-reactivity with *B. jararaca* venom when tested by different immunological methods (data not shown). Then, the cross-reacting molecules were removed by affinity chromatography (Fig. 1). Other authors have successfully used a similar approach to differentiate among the snake venoms from *Bothrops, Lachesis, Crotalus* and *Micrurus* due to their overlapping distribution in Brazil [28–31]. However, polyclonal antibodies resulting from immunization of animals vary in different matches, given the variability of animal and immunization protocols [32].

In contrast, the monoclonal antibody constitutes a valuable tool to develop methods for the identification of unknown antigens contained within a mixture of antigens, since each hybridoma is specific for a single antigenic determinant. Because of their high specificity, monoclonal antibodies are standardized reagents that can accurately point out differences in the same or in different molecules, making them important tools in the basic research, immunodiagnosis and clinical studies [33]. Nakamura et al. [34] purified a toxin from *Trimeresurus flavoviridis* venom (habutobin) and produced monoclonal antibodies that detect habutobin levels in plasma of different animals. Later, Malli and et al. [35] produced monoclonal antibodies against purified toxins from the venom of the spider *Cupiennius salei* and used it to detect venom in envenomated patients. Such antibodies contributed to an increase in technical specificity of antibodies as immunodiagnostic tools and made it possible to discover the identity of the offending animal [35].

In our study, we showed that monoclonal antibodies raised against BthTx-I are capable of distinguishing between *B. jararacussu* and *B. jararaca* venoms from different regions, as well as other Bothropic venoms (Table 1). In Brazil, there is no commercial kit available for snake venom detection. Only Australia produces a detection kit, which is based on polyclonal antibodies [36–40]. In this context, the relevance of our work is to generate tools for implementation of a detection kit focused on identifying the offending animal when two species or subspecies of clinical relevance are suspected.

The variability in the composition and activities of snake venoms is reported in several studies and can be seen at various levels including ontogenetic, geographic, sexual, inter and intra-specific [41–44]. In this context, ontogenetic changes in venoms are found in most studies that deal with variability [45, 46]. Tan et al. [47] found qualitative differences in protein profiles of neonates and adults of *Notechis scutatus* venoms. Furtado et al. [48], analyzing the actions of venoms from nine species of *Bothrops* snakes, found significant ontogenetic changes in lethal, enzymatic and the blood clotting activities. Our results show that individual *B. jararacussu* venoms were recognized by monoclonal antibodies, both from adults and neonates. These results show that BthTx-I is present in the venom of *B. jararacussu* of different ages (Fig. 3). It is also important to note that Lys49-PLA_2_ is a conserved toxin present in venoms of many species of *Bothrops* snakes. Thus, a monoclonal antibody that recognizes BthTx-I may recognize the homologous molecules in other venoms, explaining our data in the ELISA assay (Table 1).

A similar profile of recognition can be observed when comparing our monoclonal antibodies with those described by Prado et al. [49]. The camelid antibody fragments (VHH) with specificity for BthTx-I and BthTx-II from *B. jararacussu* venom were selected from an immune VHH phage display library. Corroborating our results, some clones showed reactivity against venoms from *B. moojeni, B. leucurus* and *B. diporus* and did not recognize *B. alternatus* venom [49]. This last venom contains a protein with 90% homology with BthTX-I, called BaTX. Ponce-Soto et al. [18] isolated and characterized BaTX that constitutes approximately 5% of the crude venom. We suggest that this low level of BaTX in *B. alternatus* venom is the cause of the lack of recognition.

In this context, BthTx-I from the southern *B. jararaca* venom shows 100% homology with BthTx-I from *B. jararacussu* venom, and it represents 13.8% of the proteins in southern *B. jararaca* venom [19]. This could be an obstacle to spreading the use of monoclonal antibodies developed by our group to differentiate among *B. jararaca* bites. However, the BthTx-I from *B. jararacussu* venom represents approximately 40% of total proteins, the highest concentration described so far for *Bothrops* venoms [12, 13]. This difference in BthTx-I concentration between the two *Bothrops* species may influence the signal generated by mAbs in ELISA. For this reason, the dilution of antibodies preserves only *B. jararacussu* venom detection, since it is the venom with the higher level of BthTx-I among all *Bothrops* species (Figs. 4 and 5). Furthermore, accidents caused by *B. jararacussu* and *B. jararaca* are epidemiologically relevant in Rio de Janeiro State [3], where *B. jararaca* do not produce BThx-I. Thus, the use of a diagnostic kit from a mAb may be regionally important. A diagnostic kit able to discriminate between *B. jararaca* and *B. jararacussu* venoms would solve, in Rio de Janeiro, questions related to epidemiology and treatment of patients.

There is a growing interest in the standardization of the ELISA technique in the form of an immunodiagnostic test, as used in Australia. It could be used in different locations and could offer to healthcare professionals a quick diagnosis of the genus involved in the accident and the amount of injected venom. Moreover, it is noteworthy that the data on snakebite are quite fragmentary because of the lack of information and recorded data. Therefore, the development of a regional diagnostic kit for identification of snake venoms could improve the treatment of snakebite victims and contribute to epidemiological studies.

## Conclusion

Our results demonstrate the potential of BthTx-I as a biomarker molecule to be used for antibody production (monoclonal and polyclonal) and posterior specific detection of *B. jararacussu* venom. These antibodies may be an important tool for the development of a detection kit that differentiates between venoms from *B. jararacussu* and *B. jararaca,* especially in Rio de Janeiro where both species are epidemiologically relevant.

## Abbreviations

BthTx-I: Bothropstoxin-I
mAbs: monoclonal antibodies

## References

[1] dos Santos MC, Gonçalves LRC, Fortes-Dias CL, Cury Y, Gutiérrez JM, Furtado MFD (1992). A eficácia do antiveneno botrópico-crotálico na neutralização das principais atividades do veneno de *Bothrops jararacussu*. Rev Inst Med Trop São Paulo.

[2] Luna KPO, da Silva MB, Pereira VRA (2011). Clinical and immunological aspects of envenomations by *Bothrops* snakes. J Venomous Anim Toxins Incl Trop Dis.

[3] Melgarejo AR, Cardoso JLC, França FOS, Fan HW, Málaque CMS, Haddad (2003). Serpentes peçonhentas do Brasil. Animais peçonhentos no Brasil: Biologia, clínica e terapêutica dos acidentes.

[4] Maruyama M, Kamiguti AS, Cardoso JLC, Sano-Martins IS, Chudzinski AM, Santoro ML (1990). Studies on blood coagulation and fibrinolysis in patients bitten by *Bothrops jararaca* (jararaca). Thromb Haemost.

[5] Sanchez EF, Freitas TV, Ferreira-Alves DL, Velarde DT, Diniz MR, Cordeiro MN (1992). Biological activities of venoms from South American snakes. Toxicon.

[6] Brazil V (1911). A defesa contra o ofidismo.

[7] Dias da Silva W, Guidolin R, Raw I, Higashi HG, Morais CP, Lima JF (1989). Cross-reactivity of horse monovalent antivenoms to venoms of ten *Bothrops* species. Mem Inst Butantan.

[8] Oshima-Franco Y, Leite GB, Silva GH, Cardoso DF, Hyslop S, Giglio JR (2001). Neutralization of the pharmacological effects of bothropstoxin-I from *Bothrops jararacussu* (jararacuçu) venom by crotoxin antiserum and heparin. Toxicon.

[9] Zamunér SR, da Cruz-Höfling MA, Corrado AP, Hyslop S, Rodrigues-Simioni L (2004). Comparison of the neurotoxic and myotoxic effects of Brazilian *Bothrops* venoms and their neutralization by commercial antivenom. Toxicon.

[10] Beghini DG, da Cruz-Höfling MA, Randazzo-Moura P, Rodrigues-Simioni L, Novello JC, Hyslop S (2005). Cross-neutralization of the neurotoxicity of *Crotalus durissus terrificus* and *Bothrops jararacussu* venoms by antisera against crotoxin and phospholipase A_2_ from *Crotalus durissus cascavella* venom. Toxicon.

[11] Correa-Netto C, Teixeira-Araujo R, Aguiar AS, Melgarejo AR, De-Simone SG, Soares MR (2010). Immunome and venome of *Bothrops jararacussu*: a proteomic approach to study the molecular immunology of snake toxins. Toxicon.

[12] Kashima S, Roberto PG, Soares AM, Astolfi-Filho S, Pereira JO, Giuliati S (2004). Analysis of *Bothrops jararacussu* venomous gland transcriptome focusing on structural and functional aspects: I—gene expression profile of highly expressed phospholipases A2. Biochimie.

[13] Homsi-Brandeburgo MI, Queiroz LS, Santo-Neto H, Rodrigues-Simioni CL, Giglio JR (1988). Fractionation of *Bothtrops jararacussu* snake venom: partial chemical characterization and biological activity of bothropstoxin. Toxicon.

[14] Rodrigues-Simioni L, Borgese N, Ceccarelli B (1983). The effects of *Bothrops jararacussu* venom and its components on frog nerve-muscle preparation. Neuroscience.

[15] Lomonte B, Angulo Y, Calderón L (2003). An overview of lysine-49 phospholipase A2 myotoxins from crotalid snake venoms and their structural determinants of myotoxic action. Toxicon.

[16] Soares AM, Guerra-Sá R, Borja-Oliveira CR, Rodrigues VM, Rodrigues-Simioni L, Rodrigues V (2000). Structural and functional characterization of BnSP-7, a Lys49 myotoxic phospholipase A(2) homologue from *Bothrops neuwiedi pauloensis* venom. Arch Biochem Biophys.

[17] Soares AM, Andrião-Escarso SH, Angulo Y, Lomonte B, Gutiérrez JM, Marangoni S (2000). Structural and functional characterization of myotoxin I, a Lys49 phospholipase A(2) homologue from *Bothrops moojeni* (Caissaca) snake venom. Arch Biochem Biophys.

[18] Ponce-Soto LA, Lomonte B, Gutiérrez JM, Rodrigues-Simioni L, Novello JC, Marangoni S (2007). Structural and functional properties of BaTX, a new Lys49 phospholipase A2 homologue isolated from the venom of the snake *Bothrops alternatus*. Biochim Biophys Acta.

[19] Gonçalves-Machado L, Pla D, Sanz L, Jorge RJ, Leitão-De-Araújo M, Alves ML (2016). Combined venomics, venom gland transcriptomics, bioactivities, and antivenomics of two *Bothrops jararaca* populations from geographic isolated regions within the Brazilian Atlantic rainforest. J Proteome.

[20] Fernandes I, Assumpção GG, Silveira CR, Faquim-Mauro EL, Tanjoni I, Carmona AK (2010). Immunochemical and biological characterization of monoclonal antibodies against BaP1, a metalloproteinase from *Bothrops asper* snake venom. Toxicon.

[21] Köhler G, Milstein C (1975). Continuous cultures of fused cells secreting antibody of predefined specificity. Nature.

[22] Theakston RDG, Lloyd-Jones MJ, Reid HA (1977). Micro-ELISA for detecting and assaying snake venom and venom antibody. Lancet.

[23] Towbin H, Staehelin T, Gordon J (1979). Electrophoretic transfer of proteins from polyacrylamide gels to nitrocellulose sheets: procedure and some applications. Proc Natl Acad Sci.

[24] World Health Organization. Progress in the characterization of venoms and standardization of antivenoms. WHO Offset Public. 1981;(58):1–44. http://www.who.int/iris/handle/10665/37282. 7245916

[25] McCarthy NJ (1984). Snake venom detection kit. Med J Aust.

[26] Marshall LR, Herrmann RP (1984). Cross-reactivity of bardick snake venom with death adder antivenom. Med J Aust.

[27] Ho M, Warrell MJ, Warrell DA, Bidwell D, Voller A (1986). A critical reappraisal of the use of enzyme-linked immunosorbent assays in the study of snake bite. Toxicon.

[28] Heneine LG, Catty D, Theakston RDG (1990). Development of a species-specific ELISA for Brazilian pit-viper venoms. Braz J Med Biol Res.

[29] Audebert F, Grosselet O, Sabouraud A, Bon C (1993). Quantitation of venom antigens from European vipers in human serum or urine by ELISA. J Anal Toxicol.

[30] Heneine LG, Catty D (1993). Species-specific detection of venom antigens from snakes of the *Bothrops* and *Lachesis* genera. Toxicon.

[31] Chavez-Olortegui C, Lopes CS, Cordeiro FD, Granier C, Diniz CR (1993). An enzyme linked immunosorbent assay (ELISA) that discriminates between *Bothrops atrox* and *Lachesis muta muta* venoms. Toxicon.

[32] Nelson PN, Fletcher SM, MacDonald D, Goodall DM, Jefferis R (1991). Assay restriction profiles of three monoclonal antibodies recognizing the G3m(u) allotype. Development of an allotype specific assay. J Immunol Methods.

[33] Blottière HM, Daculsi G, Anegon I, Pouezat JA, Nelson PN, Passuti N (1995). Utilization of activated U937 monocytic cells as a model to evaluate biocompatibility and biodegradation of synthetic calcium phosphate. Biomaterials.

[34] Nakamura M, Hanashiro K, Kosugi T (1993). A modification of the ELISA-double sandwich method for estimating the concentration of habutobin. Toxicon.

[35] Malli H, Imboden H, Kuhn-Nentwig L (1998). Quantifying the venom dose of the spider *Cupiennius salie* using monoclonal antibodies. Toxicon.

[36] Trevett AJ, Nwokolo NC, Kevau IH, Seaton RA (1994). Cerebrovascular accident after taipan bite. Med J Aust.

[37] Sutherland SK, Leonard RL (1995). Snakebite deaths in Australia 1992–1994 and a management update. Med J Aust.

[38] Trevett AJ, Lalloo DG, Nwokolo NC, Theakston DG, Naraqi S, Warrell DA (1995). Venom detection kits in the management of snakebite in Central province, Papua New Guinea. Toxicon.

[39] Mead HJ, Jelinek GA (1996). Suspected snakebite in children: a study of 156 patients over 10 years. Med J Aust.

[40] Southern DA, Callanan VI, Gordon GS (1996). Severe envenomation by the taipan (*Oxyuranus scutellatus*). Med J Aust.

[41] Chippaux JP, Willians V, White J (1991). Snake venoms variability: methods of study, results and interpretation. Toxicon.

[42] Martins AM, Toyama MH, Havt A, Novello JC, Marangoni S, Fonteles MC (2002). Determination of *Crotalus durissus cascavella* venom components that induce renal toxicity in isolated rat kidneys. Toxicon.

[43] Mushinsky HR, Miller DE (1993). Predation on Water Snakes: Ontogenetic and Interspecific Considerations. Copeia.

[44] Andrade RO, Silvano RAM (1996). Comportamento alimentar e dieta da “falsa coral” *Oxyrhopus guibei* Hoge & Romano (Serpentes, Colubridae). Rev Bras Zool.

[45] López-Lozano JL, de Sousa MV, Chávez-Olortegui C, Sanchez EF, Muniz EG, Bührnheim PF (2002). Ontogenetic variation of metalloproteinases and plasma coagulant activity in venoms of wild *Bothrops atrox* specimens from Amazonian rain forest. Toxicon.

[46] Saldarriaga MM, Otero R, Núñez V, Toro MF, Díaz A, Gutiérrez JM (2003). Ontogenetic variability of *Bothrops atrox* and *Bothrops asper* snake venoms from Colombia. Toxicon.

[47] Tan NH, Ponnudurai G, Mirtschin PJ (1993). A comparative study on the biological properties of venoms from juvenile and adult common tiger snake (*Notechis scutatus*) venoms. Comp Biochem Physiol B.

[48] Furtado MF, Maruyama M, Kamiguti AS, Antonio LC (1991). Comparative study of nine *Bothrops* snake venoms from adult female snakes and their offspring. Toxicon.

[49] Prado ND, Pereira SS, da Silva MP, Morais MS, Kayano AM, Moreira-Dill LS (2016). Inhibition of the Myotoxicity Induced by *Bothrops jararacussu* Venom and Isolated Phospholipases A2 by Specific Camelid Single-Domain Antibody Fragments. PLoS One.

